# Supplementary material for: The Gender Gap Tracker: Using Natural Language Processing to measure gender bias in media
Source: PLoS One. 2021 Jan 29;16(1):e0245533. doi: 10.1371/journal.pone.0245533 (PMC7845988; doi:10.1371/journal.pone.0245533)
Supplement: S1 Appendix — (PDF) [file pone.0245533.s001.pdf]

Appendix

This appendix contains technical information about each of the processes in the Gender Gap Tracker pipeline.

A.1 Data scraping

One of the challenges to deploy a scalable web scraper is to find a standard approach that can seamlessly collect and parse HTML from the different news outlet websites without excessive customization. Toward this goal, we started with the Python newspaper3k library, which allows some generalization across the several steps in the scraping pipeline, briefly described below.

Downloading category pages: A category page is an abstraction used to identify pages that may contain article URLs, even though the category page itself may or may not be an article.

Extracting categories and articles: At this stage, we extract all the URLs from the downloaded page and analyze the URLs to determine whether they fall into one or more of the following categories:

- **Invalid URL**: URLs that are out of the domain of the target news outlet; for instance, while collecting data from cbc.ca, if we find a URL pointing to the canada.ca domain, we discard it.
- **Category URL**: Any URL within the target domain with a path smaller than five subdirectories; for instance the URL shown in Example (A1-a) is a valid category URL, whereas (A1-b) would not be considered to be a valid category URL. We found that five subdirectories captured enough articles, without extracting images or other assets.

(A1). a. www.cbc.ca/news/canada/british-columbia
b. www.cbc.ca/news/canada/british-columbia/sports/hockey/nfl/
For this stage we had to manipulate the newspaper3k library, which by default only considers URLs with one subdirectory to be a category URL.

- **Article URL**: Any URL that is from the target domain or its subdomains, but is not among the above mentioned cases, would be considered to be a candidate article URL.

Downloading and checking candidate articles: Candidate article URLs are downloaded and parsed to extract the relevant information. The URLs are processed one by one in a sequential manner rather than in parallel. This helps to maintain only a small number of active connections between the scraper and the outlet, and avoids the risk of overloading the news outlet website. We then use a set of heuristics to determine whether the downloaded content shows that it is a valid news article. The criteria include, for example: whether the article has a publish date and a title, and whether the body contains at least 100 words and five sentences. If a page passes these checks, then we consider it to be a news article and move on to the next step.

Saving article to the database: In this final step, we collect the information retrieved from the page parsing process and save it in the database, a MongoDB database. The most relevant fields we store at this stage are the outlet name, the normalized URL, the title, and the body text of the news article, as shown in the example below.

- **Outlet**: Outlet name (e.g., CBC)
- **URL**: Normalized article URL (e.g., "https://www.cbc.ca/radio/thecurrent/this-woman-went-to-the-brink-of-death-and-back-to-treat-her-depression-1.5124886")
- **Title**: Article title (e.g., "This woman went to the brink of death—and back—to treat her depression | CBC Radio")
- **PublishedAt**: Published article date (e.g., "2019-05-07T19:21:13.275Z")

- **Body**: Text of the article

To uniquely identify a web ‘document’ or a news article, we use its normalized URL. This convention is important because it allows scrapers to avoid downloading a document that has been already downloaded as well as preventing most of the redundancy in the articles saved to the database.

A.2 Quotation extraction

A.2.1 Syntactic quotes. In order to extract quotations from text, we rely on the constituency or syntactic structure extracted from the text of an article. We use spaCy’s built-in dependency parser, which is a Convolutional Neural Network (CNN) model, to identify constituency structures in sentences. Our syntactic quote extraction procedure is built upon ideas in previous linguistic work [59, 109, 110]. Examples of patterns in a syntactic or dependency parse of the text can be found in Table A3 below.

The first and main pattern that we are concerned with is captured by the clausal complement structure, represented in sentence parsing with the CCOMP tag [109]. The clausal complement of a verb is a dependent clause with an internal subject, which functions as an object of the verb. For example, in the sentence *He says that you like to swim*, the clause *you like to swim*, whether it contains the complementizer *that* or not, functions as the clausal complement dependent on the verb *says*. We extract not only the quoted piece of text, but also the reporting verb (*says*) and its subject (*he* in this example). This syntactic structure captures both direct speech (within quotation marks) and indirect speech. In its indirect form, the structure is, however, very common in English and is not specific to quotations (*He saw that you like to swim; He believes that you like to swim*). To ensure that we are capturing reported speech and not other clausal objects of verbs, we created an allow-list of verbs that typically introduce quotes, which are referred to as verbs of communication [111].

Historically, the verb *say* accounts for the vast majority of instances of direct quotation in spoken language [39]. When it comes to indirect speech, however, and especially in news articles, the range of verbs used to introduce a quote expands considerably. In many cases, the verb projects attitude towards the content of the quote, stating the writer’s endorsement or distancing from what the speaker said, a form of engagement [112]. Consider, for example, the difference between characterizing a speaker as *claiming, insinuating*, or *confirming*. The list of quoting verbs that we eventually arrived at, in Table A1, was compiled from a set of pilot experiments on a large sample of news text articles and then fine-tuned as a result of our evaluations and comparisons against manually-annotated data.

In the process of manual annotation, we found that some verbs like *warn* tend to be used as a quotation verb consistently. Other verbs such as *estimate* may be used either for quoting (e.g., in *The Minister estimated that it would take 10 years to build the new transit line*) or in a different context (e.g., *It is estimated that Canadians toss out 25 per cent more trash over the holidays*). We decided not to include verbs that have this dual function in our list of quoting verbs to enhance accuracy. This, of course, results in a trade-off with the system’s recall (quotes that use this specific category of verbs will be missed).

Table A1. List of verbs that introduce quotes.

**acknowledge, add, address, admit, announce, argue, believe, claim, conclude, confirm, continue, declare, describe, ensure, estimate, explain, find, indicate, inform, insist, note, point, predict, provide, release, reply, report, respond, say, state, suggest, tell, testify, think, tweet, warn, write**

The second syntactic structure involved in quotative contexts features the prepositional phrase *according to*. Quotations that use this structure are usually of one of two types: following or preceding *according to*. In (A2-a), we see an example where *according to* precedes the content of the quote (*surveys conducted* . . .). The second example, (A2-b), shows the construction with the content of the quote at the beginning, before the quotative *according to* and before the name of the speaker of the quote. Neither example is currently captured by the quote extractor, because the parser does not extract this structure reliably, but we are working towards new development in this aspect.

(A2). a. According to Hui, surveys conducted by Altus Geomatics on June 5 and June 29 of this year showed that the shoring had not experienced any additional movement, proving that the site was stable.
b. This upcoming construction couldn't have been completed during the bridge's replacement because the money wasn't available at the time, according to Halifax Water spokesperson James Campbell.

Other syntactic structures involving quotative particles exist in English, but they tend to be characteristic of spoken or informal language, and are thus unlikely to appear in news articles. Such is the case of the quotative structure *be like*, as in *He was like, "I don't believe you"* [39, 113]. When we do find such structures, it is because the writer of the article quotes somebody using them. For example, a news article (https://www.cbc.ca/news/canada/london/covid19-symptoms-1.5536554) quotes the interaction a woman had with a health worker, in her own words: *"He was like 'no I don't think you have all the symptoms and we're low on swabs,'" she said. "I told him I had to get tested and he was like 'no you're fine you have to go home, you have food poisoning.'"* The reporting by the writer of the article uses *said* as the reporting verb. It is only the reporting of the reporting by the woman that uses *be like*.

The database record for each extracted quote consists of several fields, including the quote content, its speaker and verb as well as the character indices of each of these text spans within the news text document. An example of the extracted quotes can be found in Fig. A.1.

Fig A1. A sample JSON record for an extracted quote in our database.


```
{
        "speaker": "Anna Pippus",
        "speaker_index": "(755,766)",
        "quote": "The federal government should use policy tools to
encourage Canadians to eat a more healthful diet based more on plants",
        "quote_index": "(634,753)",
        "verb": "writes",
        "verb_index": "(767,773)",
        "quote_token_count": 20,
        "quote_type": "CSV",
        "is_floating_quote": false
    }
```

A.2.2 Floating quotes. When multiple quotes by the same speaker are present in a news article, it is often the case that only one quotative structure is used, with subsequent quotes receiving their own sentence or sentences, all in quotes, as in Example (2) in the main text of this article.

Detecting floating quotes is not possible just by using syntactic patterns as described in the previous section. In a pilot experiment, where we performed syntactic quote extraction on 10 news articles, we found 21 floating quotes (from a total of 95 quotes) which were not captured syntactically. Inspired by Krestel et al. [59], we took a regular expression approach to capture floating quotes.

In order to elaborate on the challenge of detecting floating quotes, we use the following notation to describe different patterns of quotation in text:

- V: Verb
- S: Speaker
- C: Content of the quote
- Q: Quotation mark

We manually investigated 10 news articles and found every false negative of the syntactic quote detection procedure. Each observed pattern and a representative example are shown in Table A2.

Table A2. Examples of floating quote types and their context.

Pattern	Example
QCQSV. **QCQ**	"it feels like we're living our worst nightmare" Kim told CTV News. **"The fact that we are being accused. . ."**
QCQVS. **QCQ**	"Very, very frustrated," said George. **"The money is legitimate."**
SVC. **QCQ**	Yelich claimed Monday the numbers are premature. **"Our government has invested more in education than any other provincial government in Ontario. . . ."**
**QC(Q)**. QCQVS	**"So, what you have is people who are trying to come to conclusions with lousy information**. [. . .]. "We want people to be able to make good decisions with the right information and have their voices heard," Hinderks added.
**QCQ**. SVC	**"The Canadian Hockey League has had a concussion protocol for a long time, probably close to 15 years we've been following this, so we've done a pretty good job of that, we feel."** Hamilton said that as part of the leagues' concussion protocol, the CHL has a doctor based in Ontario that reviews players' testing results any time they have symptoms.

We successfully designed a procedure to capture the three most frequent floating quote patterns: QCQSV.QCQ, QCQVS.QCQ, and SVC.QCQ. In order to find such floating quotes, first we find all syntactic quotes and determine their type based on the placement of the speaker, verb, and content, and whether the content has quotation marks around it. In a second pass, we check the beginnings of all sentences in the text and look for any sentence that begins with a quotation mark. If a sentence starting with a quotation mark is not already part of a syntactic quote, and there is a QCQSV, QCQVS, or SVC quote in the previous sentence, then it is considered to be a potential floating quote. If the same sentence or any of the next five sentences end with a quotation mark, we consider the intervening sentences to be a continuation of the floating quote, and attribute the speaker of the previous syntactic quote to this entire text span (we set the threshold of five sentences empirically).

The floating quote procedure also deals with faulty output from the syntactic quote procedure. Example (A3) illustrates a mistake made by the syntactic quote extraction procedure; the text in bold is extracted as a quote, with *I* as the speaker and *think* as the verb. As human readers, when we look at the broader context, we find that the whole sentence is in quotation marks.

To correct for this type of error, after running the syntactic quote extraction, we remove any captured quote that has a start and end quotation mark, but whose speaker and verb spans fall inside those quotation marks. This correction procedure not only removes some erroneously extracted quotes, but also helps the floating quote procedure, because such sentences are considered to be potential floating quotes.

(A3). "Those people that don't feel comfortable buying cannabis now are going to hop on and come in and I think **it's just going to increase sales volume come October."**

A.2.3 Heuristic quote extraction. In evaluations of the quotation extraction system, we found that the purely syntactic approach fails from time to time, partly due to failures in the dependency parsing. To improve the results in version 5.1, and with input from students who participated in a capstone project, we included a heuristic quote extraction process. Students participated in this project as part of the Master in Data Science—Computational Linguistics program at the University of British Columbia. Students were: Yanlin An, Danyi Huang, and Nilan Saha.

The example below highlights the need for heuristics in our quote extraction process. In this example, the speaker Lily Pallot is correctly detected by the syntactic quote rules, but the extracted quote does not span all the way back to the beginning of the sentence—in versions 4 and earlier, only the portion in bold was captured by our system. It appears that long sentences with multiple clauses separated by commas tend to break the dependency parse in spaCy, leading to a fragment of the quote being captured.

(A4). "I didn't know too much about Greta, I just heard her in the news, **but when I heard about this event I learned a bit more about her, and I am just absolutely like amazed by not only her passion, but her courage as well**," Lily Pallot told CTV News Edmonton.

To capture the entire quote span, we use either sentence or quotation boundaries to decide where a quote starts or ends. The boundaries of a quote are marked by the presence of a period or quotation marks (on either end). Just as before, we use a syntactic rule that captures a subject (speaker), verb and the quote's content, but this time, we add an additional rule that grabs all the tokens forward or backward of an encountered quotation until the opposing boundary (either a period or a quotation mark) is encountered. This allows us to capture arbitrarily long quotes with any number of comma-separated clauses.

To attribute a quote to a speaker, the heuristic method first locates a verb within the given quote span between the sentence boundaries that belongs to the list of quoting verbs (in a similar way to the syntactic rules). If a proper noun subject is found that is syntactically dependent on the verb, it is attributed as the speaker of the quote. For reasons of computational efficiency, we can only check a finite distance forward or backward of a quoting verb for dependencies, so a threshold distance of five tokens was chosen empirically in this step. We check for sentence boundaries (i.e., a period), so that speakers from a neighbouring sentence are not mistakenly attributed to a verb in the given sentence.

We designed some additional failsafes based on empirical judgment to prevent the heuristic rules going astray in anomalous instances. For instance, we ignore quotes that are less than four words long. Typically, these short clauses are not really quotes—when such clauses are captured between quotation marks, they imply that the writer of the article is distancing themselves from a piece of text that they do not directly endorse (i.e., so-called "air quotes" or "scare quotes"). Or, they could simply be fragmented clauses that are part of a larger sentence. We also cap the upper limit on quote length at 100 words, to ensure that anomalous cases with an arbitrarily large number of clauses are not accepted as quotes.

Once the heuristic rules are applied, it is important to note that there could be many duplicate quotes extracted for each article. This is because the heuristic rules also check for subject-verb relationships using the sentence's syntax, so it is likely that some of the quotes extracted this way overlap with the syntactic quotes (either partially or fully). For this reason, we include a deduplication step at the end, in which we check for a quote's span indices—if there is an overlap with another quote's span, we only keep the quote with the longer span.

We observed through our testing (using a threshold distance of five) that adding a heuristic quote extraction step with deduplication not only improves our system's precision and recall (when compared to earlier versions of the Tracker), but also helps reduce redundancy of storage by eliminating duplicate or fragmented quotes.

A.3 Identifying people and predicting their gender

A.3.1 Entity recognition and clustering. We employ the Python library spaCy (https://spacy.io/), which provides a variety of industry-strength natural language processing tools

with a solid grounding in research. We use spaCy for tokenization, parsing, and NER. Based on our initial tests, the NER component has a very high accuracy when identifying entity mentions, especially when using larger pretrained models. (After experimenting with several models, we picked the en_core_web_lg model.) The algorithm is fast and reliable; like any alternative, it nevertheless makes errors in capturing some of the mentions or tagging entity types (e.g., ORG (organization) instead of PERSON). Fig. A2 shows a snippet of an article and specific entities that spaCy identified. For clarity, just the PERSON, ORG and GPE (geopolitical entity) tags are displayed. It is clear that the model quite reliably distinguishes between entities that are people and others that represent places or organizations—this is important for downstream tasks such as attributing quotes to a human speaker.

Fig A2. Sample text with named entities identified by spaCy.

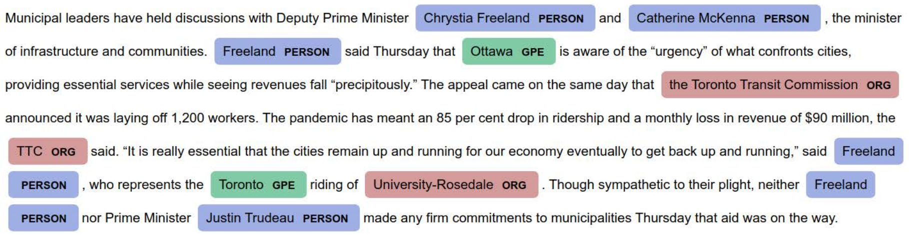

After performing NER on a document, we consider only the named entity mentions that were tagged as PERSON, and continue with coreference analysis to unify all mentions of an entity in the article. A coreference analysis algorithm takes a text and returns clusters of text spans that refer to the same entity. For people mentioned in a news article, these text spans may be: pronouns, partial or full names, or noun phrases that refer to someone without naming them, e.g., 'a Toronto mom'. Some of these references may be identified by the NER module, but certain references, such as pronouns and connections between mentions, lie outside the scope of NER.

In order to apply coreference analysis to our texts, we used NeuralCoref https://github.com/huggingface/neuralcoref), a state-of-the-art neural network coreference resolution algorithm, which is available as a Python library compatible with spaCy. The output of NeuralCoref, when applied to our text, is a set of clusters populated by different text spans that may or may not appear in our list of named entities. Our coreference analysis algorithm merges the outputs of spaCy's NER and NeuralCoref to create a set of clusters that correspond to each person, mapping all mentions of a person to one canonical name.

In order to match the coreference clusters with named entity mentions extracted earlier, we align them based on the character spans annotated by spaCy. The named entity mentions that cannot be assigned to a cluster are considered singletons, i.e., entities that are mentioned only once in the text and are not part of a cluster. After this step, we still need to merge some of the clusters because the coreference analysis algorithm is not perfect; it does not combine all mentions of a named entity within a given document, especially if the entity is mentioned in clusters that are far from each other within the text. Here we apply our domain knowledge about human names in order to find clusters pointing to the same person. There are different situations where we merge two named entity clusters: when there is an exact match, a partial match, or a three-part match.

Exact match. Due to computational cost, the coreference clustering algorithm may not be able to track mentions of a specific person in a long text, and sometimes two full names that are exactly the same fall into separate clusters. The coreference clustering function has two parameters specifying the distance between two mentions in order for those mentions to be combined (max-dist and max-dist-match). We experimented with these parameters and found that, even with large values for these, some mentions referring to the same entity would fall into separate clusters. We assume two full names that match exactly are referring to the same person and therefore always combine their clusters.

Partial match. Another common case where we consider cluster merge is when some mentions in the text consist of only the first name or last name of an entity. For example, in

the same article we may have three different clusters for *Justin Trudeau*, which should be merged into one:

- Justin Trudeau: [Justin Trudeau, He, Trudeau]
- Justin: [Justin, He, he]
- Trudeau: [Trudeau, Prime Minister Trudeau]

If one named entity mention consists of two words starting with uppercase and another mention in a separate cluster exactly matches one of these two words, we assume they refer to the same entity and therefore merge their clusters. This approach may introduce some false positive errors (over-merging), for example when two members of the same family with the same last name are mentioned within an article. Based on our experiments, the occurrence of these cases is negligible.

Note that in our cluster merging process, we only apply the above merge conditions to the cluster representatives. The representative of a cluster is one of the mentions within that cluster, chosen by the NER algorithm automatically. It is usually the longest (most complete) name in the cluster without any attached titles. We consider the longest representative among the two old cluster representatives as the representative of the new (merged) cluster. This way, we try to generate full name representatives for each cluster, which is helpful for our next step, i.e., gender recognition. The final result for the above provided example after all merges will be one cluster with *Justin Trudeau* as its representative:

- Justin Trudeau: [Justin Trudeau, He, Trudeau, Justin, He, he, Trudeau, Prime Minister Trudeau]

Three-part match. In some cases, the names have more than two parts. For example if we have two clusters with representative mentions *Adam van Koeverden* (Canadian athlete and politician) and *van Koeverden*, respectively, again the two clusters should be merged. In this case, we check whether we can match a one-part or two-part named entity mention with the three-part named entity starting from the right. In other words, we align the 'X Y Z' pattern with a 'Y Z' or a 'Z' pattern.

A.3.2 Gender prediction. The next step in the NLP pipeline involves gender prediction for each unified named entity (cluster of mentions). We experimented with two general methods to identify a person's gender in text: 1) taking the first or the full name of the person as input to gender prediction web services that work based on large name databases; and 2) relying on gender-specific pronouns used in the context to refer to that person.

Name-based gender prediction. Web services for gender prediction can be divided into two main groups: services that use the first names (typical gender of names) and services that rely on the full name of real people. We use these services as provided, and then sometimes correct errors as we encounter them.

Genderize (https://genderize.io/) and Gender-API (https://gender-api.com/) are gender prediction services based on first names. Genderize provides 1,000 free API calls per day. Gender-API is a paid service. VIAF (https://viaf.org/) contains a database of full names and provides a variety of useful information about a real person identified by their full name. This database is large, but of course does not cover all the people mentioned in the news.

Based on pilot experiments prior to deploying V4.0 of our system, we found that the first name cache gives accurate gender results comparable to our full name service, VIAF. Evaluating over a test set of roughly 10,000 names annotated for gender by hand, we observed that the free service, VIAF, had an extremely low accuracy (less than 50%), as opposed to the paid

services (over 80%). For this reason, we stopped using VIAF and instead resort to just using the first name-based services (Genderize and Gender-API).

For a given name, the following procedure is applied to predict their gender:

1. Check internal first name cache.
2. Check internal full name cache.
3. Call Genderize and update caches.
4. Call Gender-API and update caches.

Upon retrieving the gender of a name from each of the services, we cache the result for each service separately, even if the returned gender is unknown. This is done so as to prevent further API calls for the same input to the same service. Additionally, we created a first name gender cache which is filled every time a new name-gender pair is found. Most of the names are two-part full names; when we get a hit for those in the first name cache, we obtain the result with the first query to the database. The above order of calling caches reduces the number of database queries dramatically, which results in shorter processing time. When we have to query a service (due to not having the name in one of the caches), we then resort to querying one of the paid services, namely Genderize and Gender-API. One may ask why, after checking the first name cache, we still query the Genderize and Gender-API caches separately too. The reason is that we may have queried Genderize/Gender-API for that same name before and got an 'unknown' result. Since we also store these <name:unknown-gender> tuples in the caches, checking the cache helps to avoid calling the paid services again for a previously queried name. The Gender Gap Tracker project is supported by a non-profit organization and so the NLP pipeline has been designed to be cost-effective.

The internal cache allows us to periodically correct any mistakes made by the online services, for future use. As the database grows and as we perform evaluations, we find names that are regularly mislabelled by the online gender services, so those are corrected manually and added to our cache. The cache database contains 1,787 names (810 men, 575 women, and 402 'other') at the time of writing this article.

Pronoun-based gender prediction. In a set of pilot experiments, we tried to predict the gender of a named entity with the help of the personal pronouns that fall into the same coreference cluster. In this approach, we counted the number of *he* and *she* pronouns in a cluster and labelled the gender of that cluster based on the higher frequency pronoun. However, we found that this method only covered about 15 percent of the named entities extracted from the document. This is because not all named entities necessarily come with pronoun mentions in the text. Furthermore, the errors of the coreference clustering algorithm would be propagated to our gender recognition system if we use clustered pronouns as a cue. Therefore, we decided to use gender prediction services, thus we rely on names rather than pronouns. We continue to evaluate this approach, as coreference and clustering algorithms are continually improving.

A.3.3 Author identification and gender prediction. We are interested in the relationship between the gender of an article's author and the gender of the sources they quote. To that end, we clean up the author field from the scraped data and assign a gender to the author or authors in that field, following the procedure described in the previous section. The author field needs to be cleaned because the scrapers sometimes extract extra information from the article byline in addition to author names, such as: *'Posted', 'Jul', 'am ET', 'Last Updated', 'John Last'*, and *'About The Authorjohn Lastreporter'*.

Why regular expressions are inadequate. Our initial attempts to identify unwanted fields involved the use of regular expressions to catch predictable patterns (such as capitalized

combinations of words representing a person's name). However, we quickly learned that regular expressions would not be a viable option, mainly because of the sheer variety of patterns we could expect to see. A common problem with author names scraped from bylines on a web page is the presence of ambiguous words that could represent either a person or an organization. We observed that certain articles do not contain any human author names, but simply name a newswire organization (e.g., *Canadian Press* or *Associated Press*). Other articles have authors such as *Jordan Press*, which could be names of organizations, but are actually names of people. There are also many instances of author names with initials occurring in various positions (*J. Kelly Nestruck, S.R. Slobodian, Robert D. McFadden*), making this pattern matching problem non-trivial.

Rather than attempting to design elaborate (and possibly unmaintainable) regular expressions, we chose to apply spaCy's NER module to more reliably identify names of people. First, we define a block-list of author names containing major organization names and other fields such as *'Instagram', 'File', 'Photo', 'EDT', 'World'*, etc. Words in this list are chosen such that they are extremely unlikely to ever double up as the name of a person (for example, *'Press'* is not in this list). Each name in an article's author list is checked for membership in the block-list, and if so, the entire value is removed. Next, a spaCy NER pipeline is applied to the author field to extract only those entities tagged as PERSON. Lastly, a deduplication step is applied to ensure that all author names are unique in each field. In the example shown above, the field (*'John Last', 'About The Authorjohn Lastreporter'*) might yield two people's names due to spaCy capturing instances of capitalized words as names (*'John Last'* and *'Authorjohn Lastreporter'*), but it is clear that one name is just a subset of the other. The results from this author name-cleaning pipeline are then used as input to the gender prediction component, for which evaluation results are provided in the Evaluation section in the main part of the paper.

Human-inspected author names. We observed that even spaCy's large language model failed to identify certain non-Western names (e.g., *Farai Mutsaka, Ranjeetha Pakiam*) and names with English common nouns (e.g., *Hope Yen, Penny Smoke, Patience Haggin*). This is largely because spaCy's language model was trained on data from the web, so it mostly learns commonly found name patterns on the web. To deal with this, we developed a hand-curated list of name patterns by manually inspecting more than 800 human author names found in our data and noting which of these names were missed by spaCy's off-the-shelf language model during NER. The missing names are stored in a structured pattern list and are used to augment the spaCy language model's named entity rules, thereby improving our accuracy. An example of the named entity pattern rules used in this pipeline is shown in Fig. A3.

Fig A3. Example named entity patterns for author names used to augment spaCy's language model.

```
{"label": "PERSON", "pattern": "Penny Smoke"}
{"label": "PERSON", "pattern": "Hope Yen"}
{"label": "PERSON", "pattern": "Terray Sylvester"}
{"label": "PERSON", "pattern": "Morgan Black"}
{"label": "PERSON", "pattern": "Jordan Press"}
{"label": "PERSON", "pattern": "Morgan Campbell"}
{"label": "PERSON", "pattern": "Farai Mutsaka"}
{"label": "PERSON", "pattern": "Patience Haggin"}
{"label": "PERSON", "pattern": "Premila D'Sa"}
{"label": "PERSON", "pattern": "Cara Rosenbloom"}
{"label": "PERSON", "pattern": "Ranjeetha Pakiam"}
```

A.4 Evaluation

We first describe a pilot annotation (for a small collection of articles) and then the main annotation phase (for a large and balanced set of news articles with clearer annotation guidelines).

A.4.1 Manual annotation guidelines. The task of annotating a news article by humans was broken down into four sub-tasks: annotating quotations, people mentioned in the article, sources mentioned in the article (people who were quoted), and the genre of the article (hard news vs. opinion).

The first component (quote annotation) was by far the most complex and time-consuming. The annotators were asked to identify each quote in a given article, as well as the speaker and quoting verb if applicable. They were required to provide the spans of each of these objects too, which they would determine from the tokenized text form of each article. In order to

evaluate the coreference clustering system, the annotators were also asked to provide the full name of the referent for each speaker, if it could be determined. Each quote object thus consists of seven attribute-value pairs: speaker, verb, quote, speaker index, verb index, quote index and reference. A list of all the quote objects for each article could conveniently be serialized in a JSON file, which is what we required of the annotator. All the other components of annotation were done in a spreadsheet. Annotating people mentioned in the article and sources—the intersection of those mentioned in the people field who were also speakers of quotations—allowed us to evaluate our named entity recognition system.

The annotation guidelines discussed all of these aspects of annotation, and we provided one fully annotated gold sample article that annotators could use for reference.

A.4.2 Pilot annotation. We began by establishing a pilot manual annotation experiment. The annotation package included a sample of eight randomly selected news articles; each news article was prepared both in the clean and preprocessed raw text format as well as in a tokenized format (with character indices for the start and end of each token in the text).

The participants were three annotators with Bachelor's degrees aged 22, 21, and 22 respectively. All three had a low level of expertise with the annotation task. They were given the annotation package (eight articles in raw and tokenized format), the annotation instructions, and one gold sample article that we manually annotated as an example.

We calculated inter-annotator agreement for quote spans. For matched quotes, the agreement in annotation of speakers and verbs was also calculated. Agreement between the quote annotation of Annotators 1 and 2 was fairly high (90-100%) except for a few anomalous cases where the agreement was only 50%. By examining the manual results that caused these outliers in inter-annotator agreement, we found that some of Annotator 1's annotations contained errors. In the annotation of people and sources, the agreement was even lower, averaging 74.5% with a range from 38% to 89%. This was surprising given that named entity recognition for people's names is not a challenging task for humans. Inter-annotator agreement between Annotators 2 and 3 was very high in all fields. For people and sources, agreement was between 92% and 100%. For quotes, agreement was comparatively lower, but still high, with an average of 89% and a range from 76% to 100%. Given these results, we decided to discount Annotator 1's annotations and have Annotator 3 take charge of the main set of articles.

A.4.3 Improvements to annotation guidelines. Based on patterns in the errors made by the annotators in the pilot annotation, two main improvements were made to the annotation guidelines: a section about JSON file structure was added, as well as a section that explained a variety of example quotes to clarify different types of quotations in news articles. The example quotes section also included edge cases and how to handle them.

Annotators who did not have experience with data or coding found it difficult to understand the JSON file format, so a detailed appendix on JSON for non-coders was added to the guidelines. This appendix includes a general introduction to JSON and an explanation of the structure of the file, as well as instructions on when to backslash-escape quotation marks within a quote. Accurately processing the encoding for quotes is of paramount importance to this project. Annotators were also given instructions on using JSON validators online to ensure that their files were well-formed. The second improvement involved adding a section with a large number of quote types, examples of each type, and sample annotations of the examples. These quote types, as included in the annotation guidelines, are shown below in Table A3, with an example of each. We reasoned that having explanations and examples of these would yield more accurate and consistent results.

A.4.4 Main annotation. For the main and final annotation, we selected 14 articles from each of the seven news outlets, for a total of 98 articles, chosen from months of recently scraped data at the time (December 2018-February 2019). We chose articles that were

Table A3. Quote types included in the annotation guidelines.

Quote type	Example
Direct quote with speaker and verb	Lorraine Hansberry said, **"Never be afraid to sit awhile and think."**
Indirect quote	She alleged that **we should never be afraid to sit awhile and think**.
Indirect quote with embedded direct quote	Lorraine Hansberry said that **we should never be afraid to "sit awhile and think."**
Quote with no verb	**We should never be afraid to sit awhile and think**.—Lorraine Hansberry
Floating quote (implicit speaker)	**"I trusted the bank."**
Quotative verb with auxiliaries	David Saikaley had been saying that **he had faulty advice**.
Unusual quotative verbs	Rachel Notley warned **not to allow the 22-week limit to drift**.
Quote with a complement after quotative verb	They said to TD Bank's ombudsman, **"Your service is wonderful!"**
Quote with a possessive and a noun	The instructor's statement that **the exam was cancelled** met with cheers.

representative of the overall statistics according to our system, i.e., contained less than 30% female and more than 70% male sources (calculated based on the latest system release at the time of annotation). The articles were picked in a way that they were distributed across different days of the week, and each was selected to have at least 3,000 characters. Annotator 3 from the pilot annotations was chosen to continue with the main annotations. By this point, she had an intermediate level of expertise with the task. As she was the only one working on the main annotations, we currently do not have any inter-annotator agreement calculated for the main data, but we did have a second person programmatically validating the annotations for errors and manually checking them.

Through this round of data annotation and post investigation of the sampled data, we discovered that news articles quote some sources in very interesting ways that make it difficult for our automatic system to detect. Some of these cases are discussed below.

Scare quotes. Scare quotes, or air quotes, use quotation marks to draw attention to or to question the material in quotes. For instance, the headline *Biologists pen letter over Alberta MLA's 'misinformation' on conservation plans* places distance between the writer and the term *misinformation*, implying that misinformation label is not endorsed. Such quotes are not true quotes in the sense we are interested in here, as a report, reproduced verbatim, of what somebody said. A human annotator can easily tell that something is a scare quote, but the system would have to be extremely sophisticated to make this judgment. We chose not to annotate scare quotes in articles.

Fictional characters. When a TV show character is mentioned in an article, does this count as a person mentioned in an article? We draw a boundary between real people and fictitious characters, but that does not necessarily translate well for an automated system. We chose not to annotate TV show characters, but, assuming that the system captures all people, we know that this would lead to the appearance that we extract more people mentioned than are present.

What counts as a quote. What counts as a quote, and how do we decide this? The 'quotiness' of a sentence often seems to lie on a continuum rather than a category that can be captured by binary scale. For example, consider the following sentences:

(A5). In a news conference, she warned Ottawa not to allow the 22-week limit to drift.
b. Ms. Notley had urged the federal government to pass emergency legislation declaring that. . .
c. Saikaley has complained to TD's ombudsman that the bank that gave him the advice

in the first place isn't doing enough to get him out of the mess.

d. He estimates medical bills have mounted to more than $60,000, mostly because of months he spent in a nursing home unable to take care of himself

It is difficult to decide which ones to tell annotators to annotate and which ones not to, and our system would certainly find it challenging to differentiate between sentences with the corresponding syntactic structure that were actually quotes, and sentences with the same structure that were not. We decided to concentrate on clear cases of quotes with the list of verbs in Table A1, which constitute the vast majority of the quotes in news articles.

The evaluation methodology and results are described in the main part of the paper.
