# Supplementary material for: The Gender Gap Tracker: Using Natural Language Processing to measure gender bias in media
Source: PLoS One. 2021 Jan 29;16(1):e0245533. doi: 10.1371/journal.pone.0245533 (PMC7845988; doi:10.1371/journal.pone.0245533)
Supplement: Attachment — Submitted filename: Response_to_reviewers_.pdf [file pone.0245533.s002.pdf]

December 16, 2020

Re: PONE-D-20-22487 submission, "The Gender Gap Tracker: Using Natural Language Processing to measure gender bias in media"

Dear Dr. Kehler,

Thank you again for the reviews of this new version of our article. We have incorporated the feedback and also made some additional changes to the article, mostly to presentation and some slight adjustment of the statistics, due to an improvement in the latest version of the Gender Gap Tracker. We have not marked the changes on the tables, because it is tedious to mark each number on the tables themselves, but we have noted which tables have changed, in the margins.

We have also added some more references, as new research in this area continues to be published.

One significant change is that we have added Prashanth Rao to the list of authors. This paper was mostly written in 2019, with research from the original authors. Prashanth joined the team in January 2020, as we were working on revisions to the paper. His contributions to the Tracker have been invaluable, especially to the latest versions, the results of which we report in this latest version of the paper. All the original authors have provided written confirmation that they agree to add Prashanth. We are also submitting the official PLoS form to change the list of authors.

Best regards,

Maite Taboada (on behalf of all authors)

# 1 Editors' comments

Dear Dr. Taboada,

Thank you for submitting your revised manuscript to PLOS ONE. Upon receipt of the submission, I requested reviews from the original two reviewers; Reviewer 2 accepted but Reviewer 1 declined. Based on my own reading of the revision, I felt comfortable basing my decision on the judgment of Reviewer 2 and my own, and hence opted not to bring a new reviewer into the process.

Reviewer 2 and I agree that the original comments from both reviewers have been acted on in good faith, and that the paper is publishable. Because submissions that receive Accept decisions at the journal proceed straight to production, I'm taking the action of issuing a Minor Revision decision, to give you the opportunity to address the minor comments that Reviewer 2 makes in their new review, as well as any other minor modifications that you deem appropriate before the article sees print. Barring any new substantial changes, I intend to accept the revised manuscript upon a spot check. Hence it will not go out for further external review. Please submit your revised manuscript by Jan 17 2021 11:59PM. If you will need more time than this to complete your revisions, please reply to this message or contact the journal office at plosone@plos.org. When you're ready to submit your revision, log on to `https://www.editorialmanager.com/pone/` and select the 'Submissions Needing Revision' folder to locate your manuscript file.

Please include the following items when submitting your revised manuscript:

- A rebuttal letter that responds to each point raised by the academic editor and reviewer(s). You should upload this letter as a separate file labeled 'Response to Reviewers'.
- A marked-up copy of your manuscript that highlights changes made to the original version. You should upload this as a separate file labeled 'Revised Manuscript with Track Changes'.
- An unmarked version of your revised paper without tracked changes. You should upload this as a separate file labeled 'Manuscript'.

If you would like to make changes to your financial disclosure, please include your updated statement in your cover letter. Guidelines for resubmitting your figure files are available below the reviewer comments at the end of this letter.

If applicable, we recommend that you deposit your laboratory protocols in protocols.io to enhance the reproducibility of your results. Protocols.io assigns your protocol its own identifier (DOI) so that it can be cited independently in the future. For instructions see: `http://journals.plos.org/plosone/s/submission-guidelines#loc-laboratory-protocols`

We look forward to receiving your revised manuscript.

Kind regards,

Andrew Kehler, Ph.D
Academic Editor
PLOS ONE

## 2 Reviewers' comments

Reviewer #2: The revised version of the paper addresses most of the comments I had in the previous version satisfactorily. Since the authors defer the more rigorous/deeper analysis to future work (including topical analysis etc.) and the primary contribution here is the Gender Gap Tracker software, I would encourage the authors to emphasize that here they are primarily interested in demonstrating the rich analyses that the Gender Tracker would enable in the future (and some of the analyses may be strengthened further as noted in my previous comments). Finally, one point that the authors mention the response letter is that to prevent overfitting due to iterations over their rules, they ensured they always evaluated on an extension of the test set. This detail is not mentioned in the revised paper's main content – an important point which should be added.

> Thank you again for your feedback on the paper. We have indeed completed the topic analysis and are in the process of submitting a research paper about this. Preliminary results of this work have been shared online, in articles for the general public. We briefly refer to this new work in the conclusion.
>
> We have also added more information about the evaluation process and pointed out how the test set is part of the content that the Tracker normally processes, and that any true positives can be safely assumed to generalize to both old and incoming data (beginning of Section 4).
>
> Additionally, since the last version of the paper, we have completed a pilot project on text from other organizations. This was a very successful pilot, showing the adaptability of the Tracker (see the end of Section 4, Evaluation).
